# Portable perimetry devices for glaucoma patients –practicality in daily clinical practice and glaucoma expert assessment

**DOI:** 10.1007/s00417-025-07028-9

**Published:** 2025-12-04

**Authors:** Silvia Schrittenlocher, Vincent Lüke, Hanne Irle, Jithmi Weliwitage, Jan Niklas Lüke, Philip Enders, Esther Hoffmann, André Rosentreter, Jan Lübke, Sven Dinslage, Randolf A. Widder, George Kong, Algis J. Vingrys, Claus Cursiefen, Thomas S. Dietlein, Alexandra Lappas

**Affiliations:** 1 Department of Ophthalmology, University of Cologne, Faculty of Medicine and University Hospital Cologne, Cologne, Germany; 2 University of Cologne, Institute for Medical Statistics and Computational Biology (IMSB), Cologne, Germany; 3 Faculty of Medicine, University of Mainz, Mainz, Germany; 4 Faculty of Health, University Hospital Wuppertal, Wuppertal and University of Witten/Herdecke, Witten, Germany; 5 Faculty of Medicine, University of Freiburg, Freiburg, Germany; 6 Augenzentrum Bad Tölz, Bad Tölz, Germany; 7 St. Martinus Hospital Düsseldorf, Düsseldorf, Germany; 8 Royal Victorian Eye and Ear Hospital, Melbourne, Australia; 9 Centre for Eye Research Australia, Royal Victorian Eye and Ear Hospital, Melbourne, VIC 3002 Australia; 10 Faculty of Medicine, Dentistry and Health Sciences, Department of Optometry and Vision Sciences, University of Melbourne, Melbourne, Australia; 11 Center for Molecular Medicine Cologne (CMMC), University of Cologne, Cologne, Germany

**Keywords:** Glaucoma, Visual field, Virtual reality perimetry, Tablet, Melbourne rapid fields

## Abstract

**Purpose:**

To assess the practicality of two novel perimetry devices for glaucoma in daily clinical practice: a head-mounted virtual reality headset and a tablet-based perimeter. Both were compared to conventional bowl perimetry and glaucoma experts qualitatively assessed the results.

**Methods:**

The study included 363 eyes from 199 patients. All patients performed two perimetry examinations with one or both eyes: standard automated perimetry on a conventional bowl perimeter (CBP; Octopus 900, Haag-Streit) and subsequently one of two novel perimetry methods: virtual reality perimetry (VRP; n=100 patients; “PalmScan VF2000” MicroMedicalDevice) or tablet-based perimetry using the *Melbourne Rapid Fields* application (MRF; n=99 patients; Glance Optical Pty.Ltd.). Additionally, a panel of 10 glaucoma experts was asked to evaluate the new methods.

**Results:**

There was a very high correlation between VRP and CBP for mean deviation and pattern standard deviation (ICC 0.956 and 0.825, respectively). The correlation was moderate to high with the tablet-based perimetry using the MRF application (0.832 for mean deviation and 0.566 for pattern standard deviation). 74.6% of the surveyed glaucoma experts would recommend a follow-up examination with VRP whereas only 47.1% favored a follow-up examination with MRF.

**Conclusion:**

Both novel tests closely corroborated the standard bowl perimetry measurements. The diagnostic agreement was very high for the virtual reality-perimeter and moderate to high for the tablet-based perimetry, *Melbourne Rapid fields*. Based on our questionnaire, most glaucoma experts would recommend a follow-up with either method with a strong preference for the virtual reality head-mounted device.

**Supplementary Information:**

The online version contains supplementary material available at 10.1007/s00417-025-07028-9.

## Abstract (fr)

**Key messages:**

*****What is known***:**

- Perimetry in glaucoma diagnostic is an important examination with half-bowl perimetry being the standard device method.

*****What is new***:**

- Portable perimetry devices, like virtual reality-headsets and tablet-based applications, show good diagnostic accuracy compared with standard half-bowl perimeter.
- Portable perimetry devices offer novel test modalities that deliver favorable user experience and advantages regarding comfort, flexibility and the option to perform telemedicine.
- Glaucoma experts considered the portable devices appropriate and even more suitable than the half-bowl perimeter.

## Introduction

Glaucoma is worldwide one of the most important causes of irreversible blindness. It is characterized by permanent loss of retinal ganglion cell leading to visual field damage. Perimetry is an essential examination for diagnosis and long-term monitoring of functional damage in glaucoma. Standard automated perimetry is currently the most common method to evaluate visual field defects. As a subjective testing method, however, it causes difficulties for many patients: it requires concentration, time and a certain learning curve. Among the glaucoma follow-up examinations, visual field testing seems to be one of the least popular examinations [1]. Patients often refer to visual field testing as “time-consuming, old-fashioned and tiring” [2]. Furthermore, it needs to be performed “in-office” as it is not portable, and the cost of the device makes it less accessible to patients in rural or remote areas. It also requires the presence of trained personnel to set up and run the testing program.

New portable devices, like head-mounted devices or tablet-based software programs are promising and could address unmet needs in monitoring glaucoma patients. They offer several advantages, like flexibility regarding the place of testing, allowing even testing at home, can be self-administrated without prior training. Also, the main difference in the handling of VR-headset compared to the CBP is the possibility of leaning back to a more comfortable body posture. Similarly, when using the tablet-based perimeter, the patient is sitting in an upright posture and can sit comfortably in front of the tablet. Possible obstacles include diagnostic agreement, regulatory approval, cost and acceptance by patients and clinicians [3].

The purpose of the present study was to examine the diagnostic agreement of standard automated perimetry on a conventional bowl perimeter with two novel perimetry devices: the virtual reality perimetry headset and the tablet-based software *Melbourne Rapid Fields.* The outcomes from the new devices were compared to those obtained by a bowl perimeter with appropriate statistical methods. This was supplemented with a qualitative analysis of a survey of a cohort of glaucoma experts who assessed the usefulness and feasibility of the two novel perimetry methods in comparison to conventional bowl perimetry outcomes.

## Patients and methods

The present study was a prospective cross-sectional analysis of 363 eyes from 199 participants performed between 01/2022 and 10/2022 at the Department of Ophthalmology, University of Cologne, Germany.

All patients were tested with a conventional bowl perimeter (CBP; Octopus 900, Haag-Streit, Switzerland) and with one of two novel methods: the virtual reality headset (VRP; PalmScan VF2000, MicroMedicalDevice, USA) or the tablet-based perimetry application Melbourne Rapid Fields (MRF; Glance Optical Melbourne Ltd., Australia) using an iPAD Pro (Apple, Ca., USA). The examinations were performed consecutively, and order was not randomized. VRP-group consisted of 100 patients (178 eyes) and was tested with the VRP headset. MRF-group consisted of 99 patients (185 eyes) and was tested with the MRF tablet-based application. Inclusion criteria were patients older than 18 years, a diagnosis of glaucoma and previous experience with threshold perimetry on Octopus.

The CBP-examination was performed in a darkened room using the tendency oriented perimetry-strategy (G-pattern, 59 testing points, stimulus size III, 100ms duration). All perimetry examinations were supervised by the same clinician.

The following test specific data was analyzed: Mean Deviation (MD), Pattern Standard Deviation (PSD) reliability parameters (fixation loss, false-positive answers, false-negative answers) and duration of the examination. The study was conducted in adherence to the tenets of the Declaration of Helsinki and was approved by the local Institutional Review Board (No. 21-1502_2). All patients signed an informed consent prior to participating.

### Virtual reality perimetry strategy

The virtual reality device used in this study was the Palm Scan VF 2000^®^ (MicroMedicalDevice, MMD; Fig. 3a). The headset was disinfected before each examination and adjusted to the head of the patient with a strap. Testing was performed with natural pupils. The participant was instructed to look at the central fixation target and adjust focus for a clear image by rotating the knob on the top of the headset to correct for refractive errors. A cordless buzzer was used to poll responses whenever a light stimulus was seen. Most patients took off their glasses for testing, except for one highly myopic patient −11 dpt (diopters), who kept their glasses on, underneath the headset. All patients were offered a practice run and most declined except for four who completed the practice. The reason given by patients for declining the practice run was confidence in their ability to complete the test without practice. This was likely because, during the adjustment, the fixation target was clearly visualized and the buzzer could be actuated by these patients. The device was equipped with an occluder within the system, so external patching was not required. The Palm Scan uses a smartphone (Samsung Galaxy S6) as its screen for testing.

**Fig. 1:**
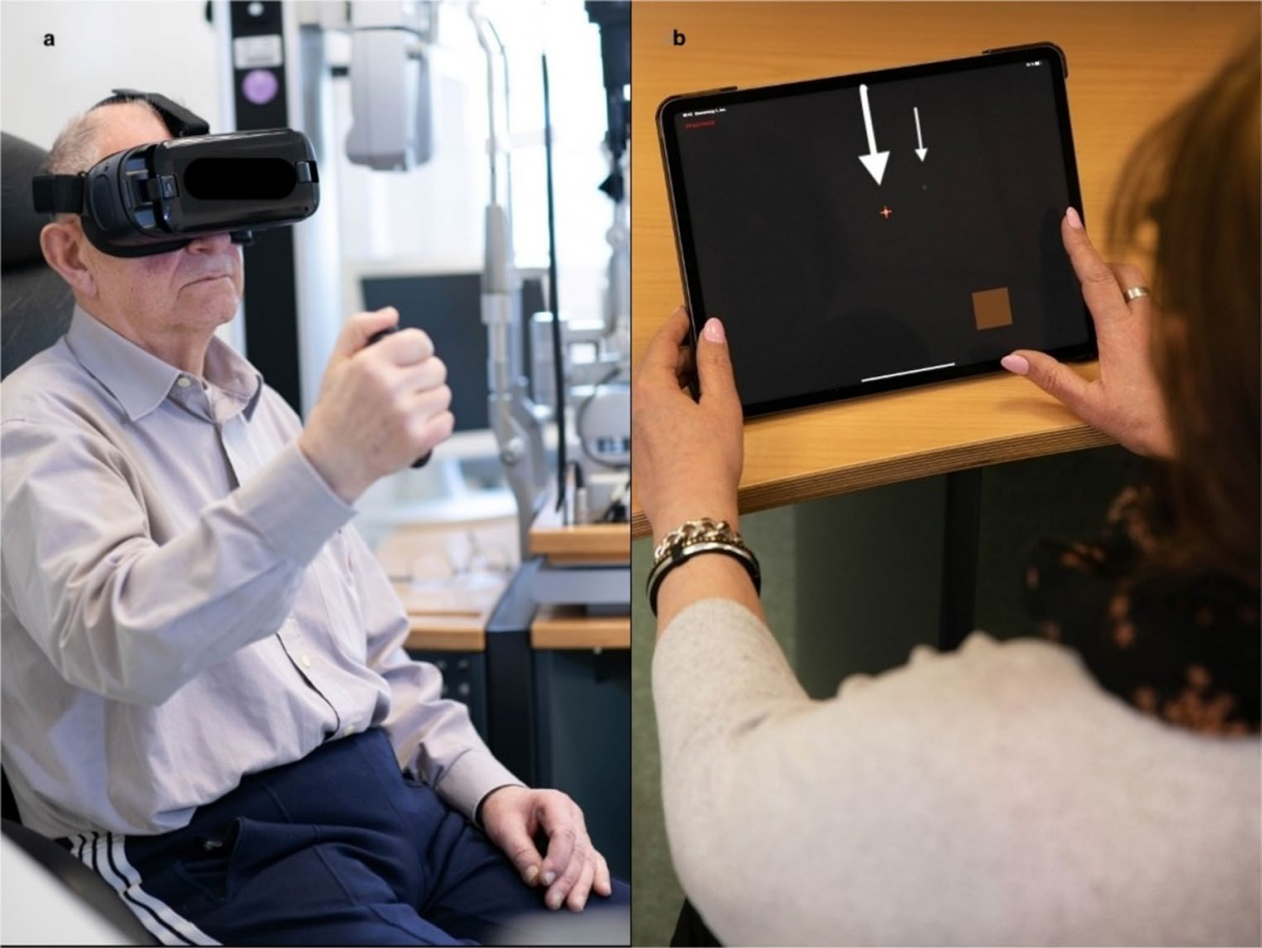
Data set presented to the glaucoma expert containing the results of the same patient: Octopus examination (2a), examination results with of one of the two novel methods (in this example MRF 2 b, c) and optic nerve OCT (2 d, e)

We used the central 24 − 2 threshold test (Interactive Fast Threshold; Fig. 1). Background illumination was 30 apostilb and the device could produce a maximum 34 decibel attenuation for a 200 ms spot. Fixation monitoring implements a Heijl-Krakau blind-spot monitor during the central fixation phase, presenting a stimulus 8–10 times in the blind-spot location.

Compared to the HBP, the VR headset offers a more ergonomic testing environment and allows for positional adjustments, such as leaning back, to improve patient comfort.

### Melbourne rapid fields strategy

**Fig. 2:**
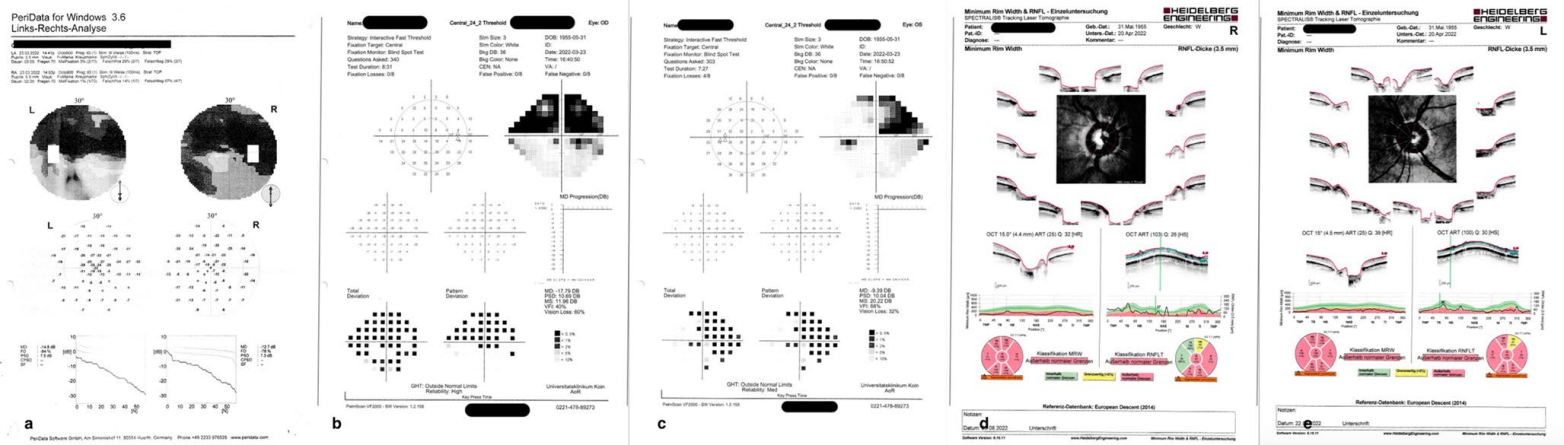
Photograph of the examination with the virtual reality-perimeter device (**a**). The patient is wearing the head-mounted device and holding the cordless buzzer in his hand. Photograph of the examination with the tablet-based perimetry application *Melbourne Rapid Fields* (**b**). The patient is sitting in front of the tablet, which is positioned on a table. The patient is fixating the red fixation cross (thick arrow) and taps the tablet surface with the finger, when perceiving the testing point (thin arrow)

**Fig. 3:**
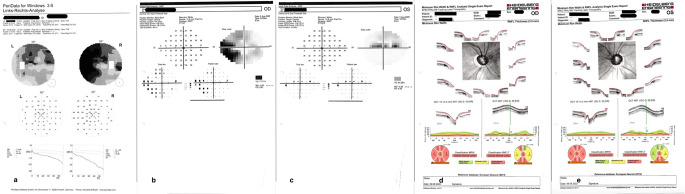
Data set presented to the glaucoma experts containing the results of the same representative patient: Octopus examination (1a), examination results with of one of the two novel methods (in this example VRP 1b, c) and optic nerve OCT (1 d, e)

MRF thresholding was conducted using an iPad PRO tablet (Model A2228, Apple, Ca. USA) and the 24 − 2 test pattern (Figs. 2 and 3b). This grid is similar to the standard 24 − 2 grid but has 4 extra test points in the foveal region. Testing was performed with natural pupils. Patients were instructed prior to testing not to move their head during the test. The examiner ensured that the patient was seated comfortably at a table with the tablet at approximately 13 inches. Care was taken to ensure that the tablet screen was not tilted with respect to the viewing plane as tilt has been shown to reduce target luminance and contrast. The tablet surface was wiped clean after every examination. All participants performed the “quick practice” mode on one eye for familiarity before performing the examination. The height of the chair and of the table was adjusted as needed. Testing was performed in free space with no constraints to head movement apart from an initial check at the start to ensure the proper viewing distance. The examiner ensured that this viewing distance was maintained during the test by sitting next to the patient. The tablet luminance output was set to maximum by the examiner before starting the test and MRF software then controls light output to provide a 30 dB testing range on a 5 cd/sq.m background. Patients were asked to wear their habitual reading glasses (single vision, bifocal or multifocal) as required. One eye was patched at a time. Patients were instructed to tap the surface of the tablet by using their index finger or thumb whenever they saw a spot in the periphery while maintaining fixation on the central red cross. The test-program proceeds in two phases: an initial central field test (36 points tested with fixation in the center of the screen) followed by a peripheral field test (20 test points) that requires the patient to change fixation to each corner of the iPad screen sequentially, to increase target eccentricity. Fixation monitoring uses the Heijl-Krakau blind-spot method. False-positive and false- negative checks are presented throughout the test [4]. One of challenges in making comparisons of MRF to Octopus outcomes is that the MRF device scales its spots from just under Goldman Size 3 in the fovea to about Goldman Size 5 at 30° eccentricity. This scaling has been undertaken to reduce variability at peripheral locations and it should yield higher thresholds than would a Goldman Size 3 spot [5]. For this reason, we will compare the mean deviation index (MD) of the MRF and Octopus devices as these relate to the performance found in age-similar normals.

The use of a tablet for MRF also allows for more comfortable patient posture compared to the half-bowl perimeter method.

### Glaucoma experts assessment

For the qualitative analysis we surveyed a cohort of ten glaucoma experts. They were asked to compare conventional perimetry to the two novel perimetry methods based on the result outputs, the optic coherence tomography (OCT) profile of the optic nerve and the patients` age (Table 1). Every glaucoma expert evaluated 10 examination results of each novel method and compared it to the conventional method. Five glaucoma experts worked at the Department of Ophthalmology Cologne and 5 were referring ophthalmologists. One expert worked in a private practice, the other 9 worked in regional or university hospitals. Each glaucoma expert received the results of randomly selected 10 examinations, both CBP and VRP and CBP and MRF including an optic nerve OCT scan taken at the time of the visual field examination (Figs. 1 and 2). These data were hard copies sent by mail. The evaluation was in terms of a scale from 1 to 10 to indicate the level of agreement for each question of the questionnaire. Some patients performed the examination just on one eye and some examinations had to be excluded because of low reliability indices. Only fully completed questionnaires have been considered for the analysis.

**Table 1:** Questionnaire and assessment regarding the result printout of the two perimetry examinations of the same patients including a reference optic disc OCT. Each question contained a rating scale from 1 (complete disagreement) to 10 (complete agreement), except for the last two questions (11 and 12). The MD ± SD value reflects the mean average according to the rating scale from 1–10. Question 11 is separately explained in the manuscript text.

Question	Group 1 (virtual reality perimetry)		Group 2 (tablet-based perimetry)		Mean difference [95% confidence interval]
	*N* (eyes)	mean difference ± standard deviation	*N* (eyes)	mean difference ± standard deviation	
1.Do you find the perimetry results of the new method shown in the grayscale topography comparable to those of the old method at a first glance?	178	7.87 ± 2.38	181	6.52 ± 2.87	1.34 [0.79; 1.89]
2.How well does the “Mean deviation” parameter match your assessment in view of the failures?	177	8.08 ± 7.19	183	6.50 ± 2.65	1.58 [0.45; 2.72]
3.In your opinion, how well does the “pattern standard deviation” parameter match in view of the failures?	177	7.86 ± 2.11	165	6.33 ± 2.29	1.53 [1.06; 2.00]
4.In your opinion, how well does the individual cooperation parameter “Malfixation” match?	177	7.83 ± 2.69	144	5.68 ± 3.45	2.15 [1.46; 2.84]
5.In your opinion, how well does the individual cooperation parameter “false-positive” match?	177	8.67 ± 2.09	181	8.13 ± 2.46	0.54 [0.06; 1.01]
6.In your opinion, how well does the individual cooperation parameter “false negative” match?	177	8.20 ± 2.36	183	8.03 ± 2.23	0.17 [−0.30; 0.64]
7.In your opinion, was the examination time of the new perimetry method appropriate in view of the patient’s age and deficits?	174	6.56 ± 2.74	183	8.09 ± 1.73	−1.52 [−2.00; −1.04]
8.Did the new perimetry technique meet your expectations with regard to the depth of the deficits with a view to the patient’s corresponding optic disc OCT?	165	7.64 ± 2.53	173	6.95 ± 2.51	0.68 [0.14; 1.22]
9.Did the new perimetry technique fulfill the expectations regarding the topography of the deficits with regard to the corresponding optic disc OCT of the patient?	169	7.67 ± 2.39	174	6.69 ± 2.58	0.98 [0.45; 1.51]
10.Was the absence of the Bebié curve on the printout of the new procedure a hindrance to the overall assessment?	172	3.47 ± 2.90	181	4.61 ± 3.32	−1.13 [−1.78; −0.48]
12.At the next check-up, would you recommend that the patient undergo a follow-up examination using the new technique?	197	74.6% yes	183	47.1% yes	N/A

### Statistical analysis

Data were analyzed by SPSS (version 28.0.1.0, Chicago, Illinois, USA) with the level of significance set at two-sided *p* < 0.05. The normality of the data was assessed using the Shapiro–Wilk test and confirmed visually with Q-Q plots. Linear mixed-effect model was used to compare the mean differences of the parameters between the groups (VRP vs. CBP and MRF vs. CBP). To address the non-independence of measures from both eyes of the same patient, random intercept for each patient was included. The device type and relevant covariates (age, gender, eye laterality) were included as fixed effects in the mixed model. Agreement between devices for various parameters was considered with intraclass coefficients and Bland–Altman analysis. To classify the severity of the glaucoma disease, we used a modified Hoddap classification and categorized by the magnitude of the mean defect (dB) for each device as: MD of >−6 dB was classified as mild, −6 to −12 dB as moderate, and <−12 dB as severe glaucoma.

## Results

### Demographics

VRP-group identifies 100 patients who performed CBP and VRP tests whereas MRF-group identifies 99 patients who performed CBP and MRF tests. The mean age (± SD) was 69.1 ± 11.9 years in VRP-group and 66.0 ± 13.2 years in MRF-group. Group gender was 52.0% male in VRP-group and 40.4% male in MRF-group.

The distribution of the glaucoma severity stage according to the CBP testing, was in both VRP- and MRF-group respectively, as follows: mild 36.8% and 43.8% respectively; moderate 25.5% and 26.7% respectively and severe 34.5% and 29.5% respectively.

### Analysis of the perimetries

The model based estimated results of virtual reality perimetry (VRP) versus tablet based perimetry using the Melbourne Rapid Fields application (MRF) and conventional bowl perimetry (CBL) are summarized in Tables 2 and 3.

**Table 2:** Overview of the results in both groups: virtual reality perimetry (VRP)-group and tablet-based perimetry with the Melbourne rapid fields (MRF)-group compared to the conventional bowl perimetry

	Virtual Reality Perimetry group			Melbourne Rapid Fields group		
	CBP	VRP	*p*-value	CBP	MRF	*p*-value
Duration (mean [95% CI]; minutes)	2.88[2.67,3.10]	6.25[6.03,6.46]	< 0.001	2.48[2.37,2.60]	4.01[3.89,4.13]	*p* < 0.001
number of tested locations (mean [95% CI])	75[69,82]	234[227,241]	< 0.001	74[72,76]	56[54,58]	*p* < 0.001
number of fixation losses (mean [95% CI])	0.01[−0.01,0.03]	0.13[0.11,0.16]	*p* < 0.001	0.01[−0.03,0.05]	0.39[0.35,0.44]	*p* < 0.001
mean defect (mean [95% CI]; dB)	−9.76[−11.0,−8.5]	−8.77[−10.0,−7.51]	*P* = 0.069	−5.48[−6.85,−4.11]	−6.88[−8.25,−5.51]	*p* = 0.026
patter standard deviation (mean [95% CI]; dB)	4.93[4.42,5.44]	5.52[5.02,6.03]	*P* = 0.019	4.69[4.15,5.22]	7.56[7.05,8.06]	*p* < 0.001
false positive answers (mean [95% CI])	0.08[0.06,0.09]	0.03[0.02,0.05]	*p* < 0.001	0.11[0.08,0.14]	0.09[0.07,0.13]	*p* = 0.254
false negative answers (mean [95% CI])	0.12[0.08,0.15]	0.15[0.11,0.18]	*p* = 0.109	0.09[−0.02,0.19]	0.22[0.11,0.33]	*p* = 0.087

**Table 3:** Interclass correlation between VRP versus CBP (3a) and MRF versus CBP (3b). The agreement between the CBP and VRP for MD was poor in early and moderate glaucoma, but improved to good in severe disease (ICC = 0.779). PSD showed only fair agreement in mild disease (ICC = 0.405), with weaker or statistically uncertain agreement in other stages. Agreement between CBP and MRF devices for global visual field indices is poor to fair, and only becomes statistically meaningful for MD in severe glaucoma (ICC = 0.448). PSD showed no consistent or significant agreement across any severity level

a CBP vs. VRP					
Variable	Severity	ICC	95% CL lower	95% CL upper	sig
MD	Mild	0.280	0.043	0.489	0.004
	Moderate	0.328	0.029	0.567	0.001
	Severe	0.779	0.655	0.862	< 0.001
PSD	Mild	0.405	0.181	0.589	< 0.001
	Moderate	0.368	0.101	0.585	0.004
	Severe	0.508	−0.041	0.771	< 0.001
**b** CBP vs. MRF					
Variable	Severity	ICC	95% CL lower	95% CL upper	sig
MD	Mild	0.134	−0.094	0.354	0.126
	Moderate	0.082	−0.235	0.383	0.308
	Severe	0.448	0.180	0.655	< 0.001
PSD	Mild	0.043	−0.091	0.205	0.274
	Moderate	0.298	−0.089	0.606	< 0.001
	Severe	0.097	−0.080	0.313	0.076

The mean MD in VRP and CBP (−8.77[−10.0,−7.51]dB vs. −9.76[−11.0,−8.5]dB, *p* = 0.069) showed statistically no significant difference (Cohen’s d = 0.194).

The mean MD in MRF and CBP (−6.88[−8.25,−5.51] vs. −5.48[−6.85,−4.11], *p* = 0.026; Cohen’s d = 0.022) differed significantly.

Accordingly, mean PSD in MRF group differed statistically and practically to the mean PSD in CBP (7.56 [7.05,8.06] dB vs. 4.69 [4.15,5.22] dB, *p* < 0.001, Cohen’s d = 1.328). The effect of observed significant difference in mean PSD in VRP compared to CBP was small (5.56 [5.02,6.03] dB vs. 4.93 [4.42,5.44] dB, *p* = 0.019, Cohen’s d = 0.249).

The mean examination duration was slightly longer with VRP and with MRF compared to the conventional bowl perimetry (6.25 [6.03,6.46] minutes vs. 2.88 [2.67,3.10] minutes, *p* < 0.001,Cohen’s d = 2.516 and 4.01 [3.89,4.13] minutes vs. 2.48 [2.37,2.60] minutes; *p* < 0.001, Cohen’s d = 2.688).

Testing for false answers revealed no significant differences of false negative answers and of false positive answers in the MRF-group compared to CBP-group.

There was no statistical difference in false negative answers between the VRP- and the CBP-group. There was, however, a significant difference in false positive answers showed in VRP-group a small to medium effect (false negative *p* = 0.109; false positive *p* < 0.001; Cohen’s d=−0.474).

**Fig. 4:**
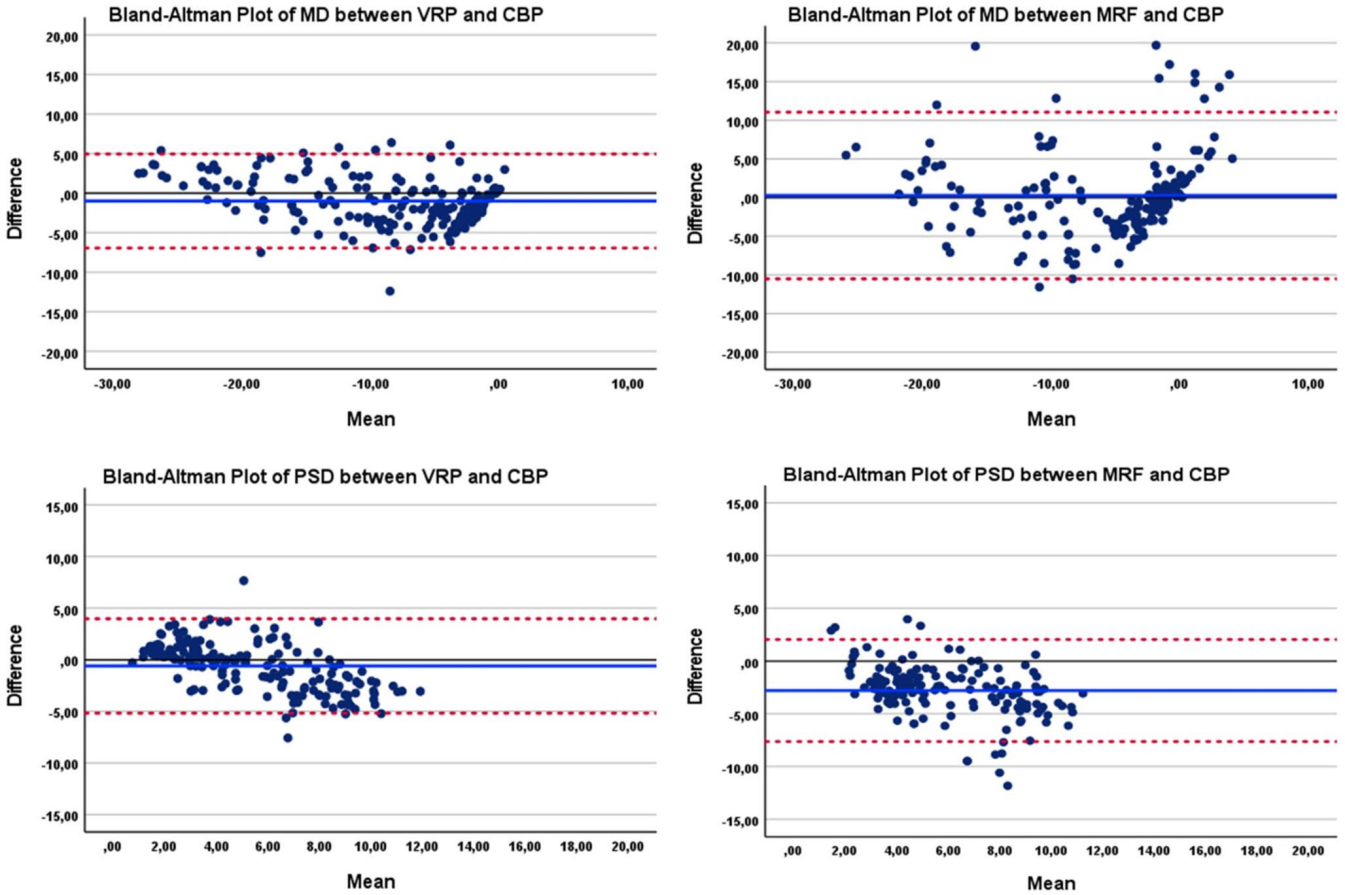
a. Bland-Altmann plot showing the diagnostic agreement of the VRP compared to the CBP by the parameter mean deviation MD (6a), by the parameter pattern standard deviation PSD (6b). In MRF-group, the plot shows the CBP compared to MRF by MD (6c) and PSD (6d). The bias (average difference) of MD in VR is −0.99 ± 3.03 and in MRF 0.28 ± 5.05. The bias (average difference) of PSD in VR is − 0.59 ± 2.33 and in MRF − 2.79 ± 2.47

The intraclass coefficients (ICC) for VRP compared to CBP were 0.956 for mean deviation (MD) and 0.825 for the pattern standard deviation (PSD) indicating a high level of concordance (Fig. 4a). In comparison, the ICC for MRF and CBP MD was 0.832 and 0.566 for PSD (Fig. 4b) indicating a moderate to high correlation for MRF and CBP parameters. The visual representation of the agreements of MD and PSD in both groups provided by Bland-Altman plots indicates a potential systematic bias of PSD in MRF-group.

### Glaucoma expert assessment

Each glaucoma expert evaluated the result between each VRP versus CBP and MRF versus CBP separately. The results of the glaucoma expert assessment are shown in Table 1. The mean and the standard deviation of each question on a scale of 1 to 10 was calculated and statistically analyzed. The table shows the results for each the VRP and the MRF evaluations. In general, all experts favored the VRP over MRF in terms of agreement compared to conventional bowl perimetry (questions 1 to 6, 10) and the optic nerve OCT (questions 8 to 9). The only exception was question number 7 regarding the test duration, which was significantly shorter in the MRF-group than in the VRP-group. Question 11 is not displayed due to limited space. This question concerned the selection of an illustration on the perimetry-printout, and was considered unnecessary for general evaluation. Some of the glaucoma experts (28.3% and 24.4% respectively) considered the illustrations of the numerical defects of the VRP examination as not vitally necessary for general evaluation. In the printout of the MRF examination 65.4% of glaucoma experts would not discard any of the illustration on the provided printout.

Furthermore, question 10 was evaluated separately. This question was meant to find out if the Bébie curve was important for the assessment of each expert or not. Did the expert need the Bébie curve to assess the perimetry result or not? Was the absence of the Bébie curve on the printout a hindrance for the overall assessment? The answer grading score was “1 for „no, not at all“ and “10 „yes, definitely“. So, if the score was low, then the expert was not bothered by the fact that the Bébie curve was NOT on the printout of the new perimetry devices.

Also, question 11 was evaluated separately. This question aimed to ask the experts’ opinion upon which representation on the printout is unnecessary. To summarize, the experts regarded the numerical total deviation map and the numerical pattern deviation map as redundant on the VR- printout. Regarding the MRF printout, the experts found all representation on the printout meaningful.

Finally, follow-up examinations with the VRP were recommended by 74.6% of the glaucoma experts whereas only 47.1% would choose the MRF for this purpose (question 12).

## Discussion

Visual field examination represents the functional testing of glaucoma and is an important instrument for glaucoma management. The results of our study show that two novel perimetry methods, the virtual reality perimetry and the tablet-based perimetry with the *Melbourne Rapid Fields* application, have a high diagnostic agreement compared to the conventional bowl perimetry (CBP).

According to the literature, Shetty et al. used the same VR-headset as in our study and reported an agreement of 1.0 in a cohort of 166 eyes [6]. Other study groups used different VR-devices from other manufacturers and reported an agreement between 0.62 and 0.86 [7–13]. As a comparison, in our cohort, we found an agreement of 0.956. The variance of these numbers could be explained by the variety of devices, the different cohort sizes, and the different study protocols. All studies compared the VRP with the Humphrey perimeter, except for Stapelfeldt et al., who compared the Octopus with the Oculus Quest perimetry in 70 eyes and reported a correlation of 0.75 [8].

Particularly in the most severe cases of glaucoma, the correlation improved compared to early or moderate stages suggesting that while MRF may be suitable for assessing advanced glaucomatous damage, its performance in detecting early or focal defects is limited, particularly when compared on global indices like MD and PSD.

Regarding the diagnostic agreement of the tablet-based application (MRF) compared to the CBP, we report a good agreement (0.832), similar to other studies that reported also good agreements between 0.80 and 0.93 [4, 14]. Possible disadvantages of the tablet-based perimetry compared to the CBP may be the smaller size of the screen compared with a full visual field dome and a smaller dynamic range of stimulus intensity [4]. Our observation on a higher rate of false positive results using the tablet may reflect this possible deficiency. The mobility of the tablet – whilst allowing a more comfortable patient posture – may add to a limited stimulus accuracy due to the smaller screen and the greater patient mobility. The performance on tablet could be improved with the use of a connected keyboard and webcam to monitor patient position (available in more recent web-based version). Also, it is to be mentioned that MRF shows a ‘learning’ improvement in dB from test 1 to test 2 [14]. It likely arises from participants becoming used to the computer interface.

Another relevant aspect is the glaucoma severity stage of the examined patients. In the MRF testing, the registered multiple shifts in fixation to the corners of the screen may result in an increased variability in threshold. Prince et al. tested the MRF in 103 patients in a low-income region in Ghana, where many patients have already an advanced glaucoma stage. Surprisingly, the authors found that MRF performed well and might be more suitable for detecting moderate to advanced cases of glaucoma than cases of early glaucoma [15]. In our cohort, we also found a slightly better correlation in severe cases than in mild glaucoma cases. Schulz et al. also reported that some early cases were not detected by MRF [16]. In contrast, Vingrys et al. validated the MRF perimetry and proved an adequate potential to detect even early defects which had MD values smaller than 3.3 dB [17]. It seems, that the validity of tablet perimetry must be further examined specifically in patients with early stages of glaucoma. The inclusion of participants across the spectrum of glaucoma severity, as defined by CBP device, is a strength of this study as it allowed evaluation across mild, moderate, and severe disease. However, the unequal distribution of severity, particularly the predominance of mild cases, especially in the MRF group may have influenced the outcomes. This aspect of the heterogenous population in different glaucoma stages might be a subject of limitations. The higher proportion of mild glaucoma could have led the MRF to classify borderline defects as “mild,” inflating this category. Thus, the observed distribution likely reflects both the underlying study population and the way early-stage defects are captured by MRF testing. The majority of the included eyes suffered from mild glaucoma stage. However, in our cohort we found a better correlation in more severe cases than in mild ones.

Concerning the glaucoma experts’ assessment, we decided to not only analyze the novel perimetry methods based on statistical parameters but also based on expert opinions. The rationale was based on the fact that perimetry is, per definition, a subjective testing method and requires an evaluation that exceeds the quantitative parameters that are provided for each test result. Although the statistical analysis formed the core of our comparison of novel versus conventional perimetry, we also included a qualitative assessment by glaucoma experts. We chose 10 glaucoma experts and used a scaling system to allow a statistical analysis of their individual evaluations. 74.6% of the glaucoma experts involved in this study were convinced by the VRP after comparing the conventional perimetries with the novel perimetries. They would recommend the patient follow up examination with the VRP, while only 47.1% would recommend further examination with the tablet based perimetry, MRF software. One exception was the duration time: hereby favorized the glaucoma experts the MRF over VRP, which shows that glaucoma experts favorize a shorter duration in a visual field examination. In our qualitative questionnaire assessment, the level of agreement of the experts on a scale from 1 to 10 of the CBP results compared to the novel methods, ranged between 7 and 9 for the VRP and 6 to 8 for the MRF. Our results showed that the expert opinions reflected our statistical results rather well.

Considering recent technological advances, we are very likely going to experience further progress in digital diagnostic tests and equipment, that will affect both patients and examiners in their daily routine. Qualitative tests of this sort might be a way to include this type of practical evaluation that is so prominent in daily practice.

One disadvantage of the novel methods was a longer test duration. The test with the VRP took substantially longer than with the CBP or with the tablet based perimetry. This delay was mainly due to the VR-headset software recommended by the manufacturer. This algorithm uses more test locations for monitoring and controlling the position of the blind spot during the examination. We observed a comparable duration between MRF and CBP. Schultz et al. also found in a cohort of 60 glaucoma patients a similar test duration between MRF and the Humphrey Field analyzer [16]. Kong et al. observed a slightly shorter duration compared to the Humphrey Filed analyzer [18]. A new “Rapid” test protocol has been developed for MRF that the maker claims is about 1.5 min faster than the protocol used in our trial and similar to SITA-faster in terms of test duration.

In a previous study we compared the acceptance of VRP and tablet based MRF perimetry in glaucoma patients. The patients were assessed with a questionnaire on their experience including test duration, need for concentration, and possible difficulties with the new examination method compared to the conventional bowl perimetry. Hereby, we observed that most of the patients would prefer the tested portable perimetry methods over conventional bowl perimetry for future regular visual field examinations, even if they tested longer [19].

One major advantage of portable perimetry devices is the possibility to perform home testing without an examiner. Portable digital devices may fit into the concept of telemedicine, providing an improved follow up-regimen. Moreover, both VRP and MRF offer a high flexibility on examining patients that physically cannot perform the examination on a CBP because of disabilities. This represents a major benefit that could lead to a stronger patient adherence and a higher testing frequency.

Camp et al. questioned in a short article the role of visual field testing in the future and discussed the role of home-testing [3]. Based on our results we are positive, that visual field testing with portable perimetry methods will grow in interest as they provide comparable results to standard bowl perimetry and are user friendly. They also may offer a major relief for ophthalmologists reducing instrument and personnel resources. All these factors have the potential to improve patient adherence and diagnostic management of glaucoma patients.

In summary, both portable perimetry devices provided a high diagnostic agreement compared to the conventional bowl perimetry. The head-mounted virtual reality device showed slightly higher agreement than the tablet-based application Melbourne Rapid Fields. Based on our questionnaire most of the involved glaucoma experts in this study would recommend follow up examinations particularly with the VRP. Portable perimetry devices could supplement our diagnostic armamentarium as they combine adequate diagnostic quality with greater patient comfort and flexibility for the detection and monitoring of glaucoma disease.

## Supplementary Information

Below is the link to the electronic supplementary material.

Supplementary Material 1
