# Supplementary material for: Portable perimetry devices for glaucoma patients –practicality in daily clinical practice and glaucoma expert assessment
Source: Graefes Arch Clin Exp Ophthalmol. 2025 Dec 4;264(4):1059–69. doi: 10.1007/s00417-025-07028-9 (PMC13002660; doi:10.1007/s00417-025-07028-9)
Supplement: Supplementary file 1 — Supplementary Material 1 [file 417_2025_7028_MOESM1_ESM.pdf]

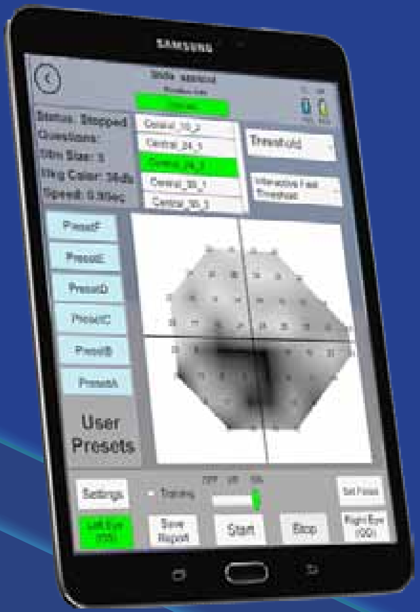

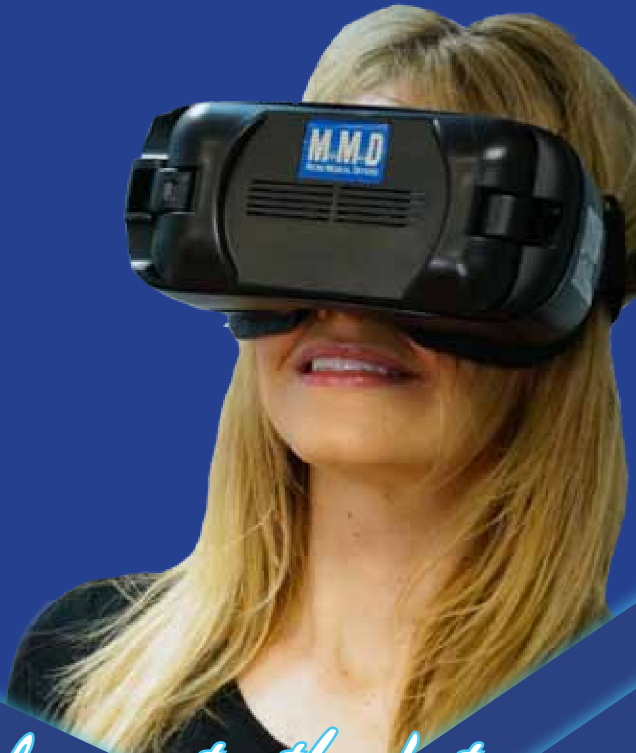

Welcome to the future of

OF VISUAL FIELD TESTING

PalmScan™ VF2000
Visual Field Analyzer

Truly Portable, Wearable Virtual Reality Perimetry
Includes 24-2, 24-1, 30-2, 30-1 and 10-2 Test Patterns

- **Portable & Battery Operated**
- **Superior Accuracy**
- **Quick Test Results**
- **Cloud Based Telemedicine**
- **Affordable**
- **Familiar Reports**

ALSO AVAILABLE FOR IN HOME PATIENT VISUAL FIELD MONITORING

M.M.D
MICRO MEDICAL DEVICES

Solutions that Fit

PalmScan
VF2000 Visual Field Analyzer

Quality handheld perimetry to aid in glaucoma diagnosis and management

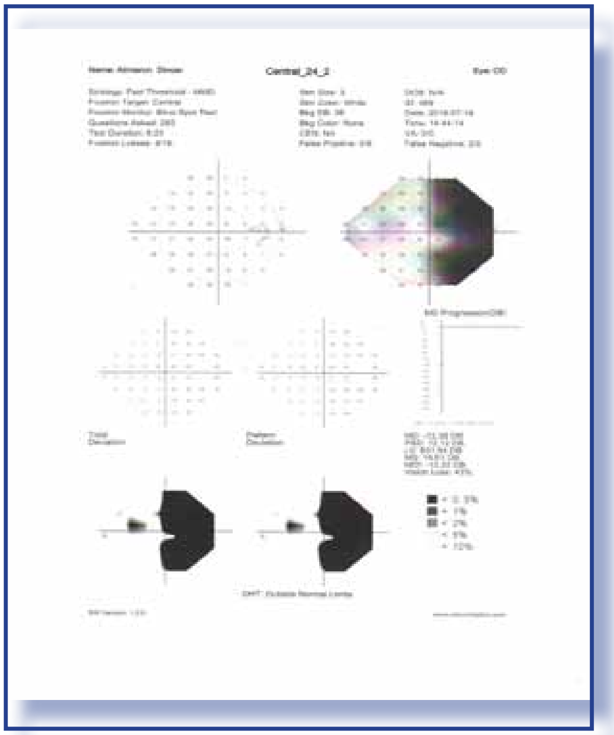

PalmScan VF2000 Visual Field Analyzer is an innovative and disruptive technology for testing of visual field defects in the medical industry. This battery operated and fully portable system allows users to perform different types of Visual Field on patients in any setting such as an office waiting room, nursing home or even in a patient's home.

For pediatric and disabled patients who cannot take the test with standard desktop equipment, the VF2000 can easily be used to complete a visual field test.

The new "Interactive Fast Threshold" algorithm quickly performs a threshold test and evaluates patient's vision in a few minutes. The system is completely independent of the ambient light in the room and you do not need a dark room to perform the test. In addition, there is no need for an eye patch to occlude the fellow eye.

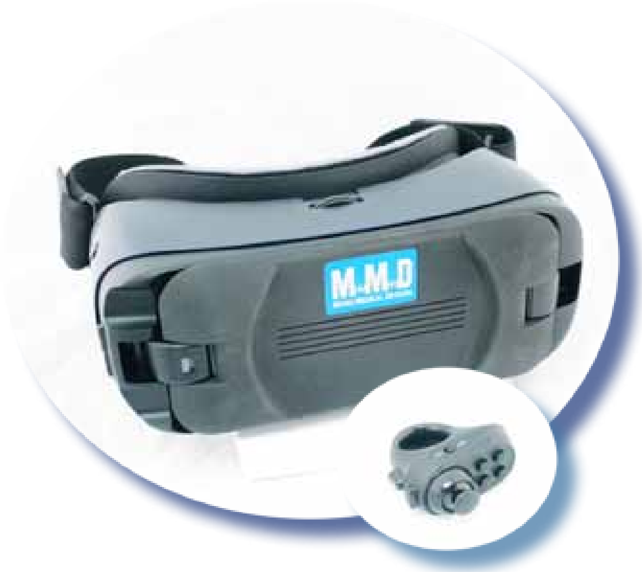

Secured Telemedicine Portal

MMD's secure Telemedicine portal gives your team immediate access to your patients reports no matter where you are. Use this portal to add new patients, archive old ones, print reports and much more all from your favorite web browser. ***Included free of charge for 12 months.**

Increase your revenue with the PalmScan VF2000 Visual Field Analyzer

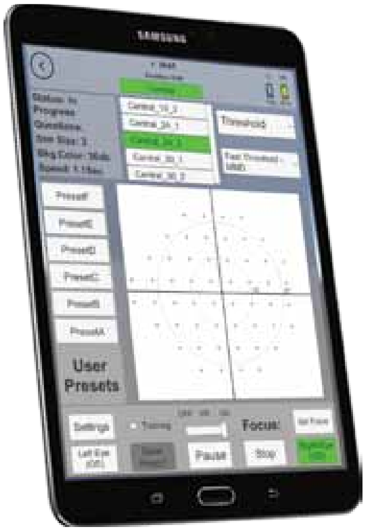

The PalmScan VF2000 Visual Field Analyzer system will help you increase your office revenue by allowing you to test patients who are unable to easily come to your office. Send your technician with this system to a local nursing home and perform full threshold Glaucoma screenings on your patients there.

The Controller app (indicated to the right) has a user friendly interface. It offers a multitude of tests for detecting Visual Field defects.

The printed report is designed to be familiar for most physicians and it includes numerical and gray scale maps for Absolute, Total and Pattern deviations. In addition, Mean Deviation progressions is also graphed so the physician can quickly determine any significant visual field changes.

PalmScan VF2000 Visual Field Analyzer qualifies for the 50% ADA Tax Credit (USA only).

PalmScan
VF2000 Visual Field Analyzer

Features

These are some of the unique features of the Palm-Scan VF2000 Visual Field Perimeter:

- Fully portable
- Fast measurements
- 70 degrees Field of view testing (+/- 35 degrees)
- 6 diopters of optical adjustment built in
- Full threshold tests
  - **Full Threshhold Fast**
  - **From Master**
  - **From Prior Data**
  - **Interactive, Fast Threshhold**
- Screening tests
  - **Single Intensity**
  - **Threshhold Related**
  - **3 Zone**
  - **Quantify Deficit**
- Ptosis tests
- Neurological tests
- Patient database and archiving
- Fixation light with Gaze Monitoring
- Ability to test one eye at a time
- Adjustable test speeds
- 6 Presets per user for your favorite con-figurations
- WiFi & Bluetooth printing capabilities
- PDF Print & EMR exportability
- Immediate web based access to captured reports
- Wireless Rapid Charger
- Bluetooth Clicker

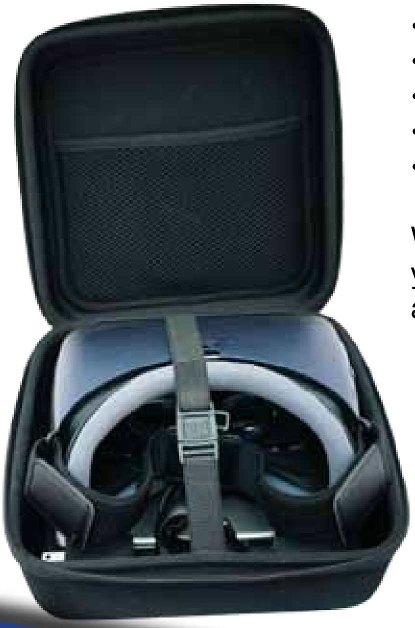

Benefits

The following are some of the many benefits of using the world's first Virtual Reality based Visual Field Analyzer:

- Highly sensitive, reliable and reproducible results
- Can be used in almost any settings
- Can be used for pediatrics patients
- Can be used for disabled patients
- Screening patients in nursing home
- Can be used to test for toxicity for certain medications and for retinal diseases
- Patient Comfort - can be used with patient in any position/orientation
- Fast measurement times

System Includes:

The following are the items that are included with the order:

- PalmScan VF2000 Virtual Reality Goggles
- Android Controller Tablet
- Clicker for patient use
- Wireless charger for the VR system
- Carry Case, Tablet Holder and Charger

With each system order an account will be setup for your office on our secure web portal with an admin account to setup other staff accounts.

PalmScan VF2000 qualifies for the 50% ADA Tax Credit (USA only).

Proudly made in the U.S.A.

Visit Micro Medical Devices at www.micromedinc.com or contact your Authorized Micro Medical Devices, Inc. Distributor.

IMPORTANT NOTICE
For your own protection, only purchase and service your Micro Medical Devices Inc. devices through an Authorized MMD Distributor.

c 2014 Micro Medical Devices, Inc. Specifications subject to change with-out notice. Micro Medical Devices, Inc. products are designed and manufac-tured under quality processes meeting ISO and CE requirements

A New Generation of Ophthalmic Convenience and Efficiency

Micro Medical Devices, Inc.
23945 Calabasas Road, Ste. 110
Calabasas, California 91302
Toll Free: 1-866-0MMD (0663)
Office: 818-222-3310
Fax: 818-337-1952
www.micromedinc.com
ISO/CE certified
Micro Medical Devices, Inc. products are designed and manufactured under quality processes meeting ISO and CE requirements
